# pVHL Mediates K63-Linked Ubiquitination of nCLU

**DOI:** 10.1371/journal.pone.0035848

**Published:** 2012-04-20

**Authors:** Jing Xue, Dan-dan Lv, Shi Jiao, Wenting Zhao, Xuebing Li, Heng Sun, Bing Yan, Li Fan, Rong-gui Hu, Jing Fang

**Affiliations:** 1 The Key Laboratory of Nutrition and Metabolism, Institute for Nutritional Sciences, SIBS, Chinese Academy of Sciences, Shanghai, China; 2 The Institute of Biochemistry and Cell Biology, SIBS, Chinese Academy of Sciences, Shanghai, China; University Health Network, Canada

## Abstract

pVHL, product of von Hippel-Lindau (VHL) tumor suppressor gene, functions as the substrate recognition component of an E3-ubiquitin ligase that targets proteins for ubiquitination and proteasomal degradation. Hypoxia-inducible factor α (HIFα) is the well-known substrate of pVHL. Besides HIFα, pVHL also binds to many other proteins and has multiple functions. In this manuscript, we report that the nuclear clusterin (nCLU) is a target of pVHL. We found that pVHL had a direct interaction with nCLU. nCLU bound to pVHL at pVHL's β domain, the site for recognition of substrate, indicating that nCLU might be a substrate of pVHL. Interestingly, pVHL bound to nCLU but did not lead to nCLU destruction. Further studies indicated that pVHL mediated K63-linked ubiquitination of nCLU and promoted nCLU nuclear translocation. In summary, our results disclose a novel function of pVHL that mediates K63-linked ubiquitination and identify nCLU as a new target of pVHL.

## Introduction

pVHL, product of von Hippel-Lindau (*VHL*) tumor suppressor gene, acts as a multipurpose adaptor protein that controls a diverse array of gene expression programs, as well as extracellular matrix assembly and microtubule-based processes by linking various target proteins to appropriate enzymatic activities [1]. Germline mutations in *VHL* can be found in patients with VHL disease, a familial tumor syndrome characterized by the development of highly vascularized tumors in multiple organs [2], [3]. Major clinical manifestation of VHL disease include hemangioblastomas of the retina and central nervous system, renal cysts and clear cell renal cell carcinoma, pancreatic cysts and tumors, as well as pheochromocytomas [2], [3]. *VHL* is also mutated in the majority of sporadic clear cell renal cell carcinoma. pVHL forms a multi-protein complex that contained elongin B, elongin C, Cul2, and Rbx1 and functions as the substrate recognition component of an E3-ubiquitin ligase complex that targets proteins for degradation [4]–[6]. The best-understood function of pVHL relates to its role that targets the alpha subunit of hypoxia-inducible factor (HIFα) for destruction [3], [7]. pVHL binds to hydroxylated HIFα and mediates HIFα ubiquitination and proteasomal degradation [8], [9]. The pVHL-mediated degradation of HIFα is involved in tumor progression as well as oxygen sensing [10]. Besides HIFα, pVHL has been found to bind to a dozen different proteins and mediates other biological processes [1], [3], [11]. pVHL has a direct interaction with atypical protein kinase C and inhibits its activities [12]. pVHL binds to hydroxylated α-chain of collagen IV [13] and reacts with fibronectin [14]. pVHL is also involved in regulation of NF-κB [15] and Wnt signaling [16].

Clusterin (CLU) is an enigmatic glycoprotein with a nearly ubiquitous tissue distribution, involved in many biological processes [17]. CLU is also believed to be involved in many pathological states including cancer [18]. It may promote cell survival or induce cell apoptosis, depending on its different isoforms. [18], [19]. The secreted form of CLU (sCLU) is present in almost all physiological fluids [20] and is considered to be a survival factor [21]. In addition to sCLU, a nuclear form of CLU (nCLU) exists. And nCLU has been found to trigger cell death in a few systems [22].

Previous study demonstrated that expression of sCLU was attenuated in cells lacking wild-type pVHL [23]. However, the effect of pVHL on nCLU is unknown. We found in our work that pVHL bound to nCLU but did not target it for destruction. Interestingly, we found that pVHL mediated K63-linked ubiquitination of nCLU and promoted its nuclear translocation. Our results reveal a novel function of pVHL that mediates K63-linked ubiquitination and identify nCLU as a new target of pVHL.

## Materials and Methods

### Cell culture and reagents

Human embryonic kidney 293T cells and human cervix cancer Hela cells were cultured in DMEM with 10% serum, penicillin (100 U/mL), and streptoMycin (100 µg/mL). Human colon cancer HCT116 cells were cultured in McCoy's 5A media (M5A) with 10% serum, penicillin (100 U/mL), and streptoMycin (100 µg/mL). pVHL antibody was from BD Biosciences (Bedford, MA). nCLU (B5), HA, Myc and His antibodies were from Santa Cruz Biotechnology (Santa Cruz, CA). Antibody against Ub(K63) (BML-PW0600-0025) was from Enzo Life Sciences (Farmingdale, NY). Beta-actin and α-tubulin antibodies, cycloheximide and MG132 were from Sigma (St. Louis, MO).

### Construction of vectors

nCLU was constructed by polymerase chain reaction (PCR) and cloned into pcDNA3.0 vector (with HA tag) or pcDNA3.1 vector (with Myc tag). Deletion forms of VHL were cloned into pCDNA3.1 vector or into pET-28a Vector (with His epitope). R-VHL was constructed through inserting VHL into pDsRed-Monomer-N1 vector between Xho I and Hind III. Glutathione S-transferase (GST)-nCLU fusion proteins were constructed by inserting PCR-generated DNA fragments encoding nCLU into pGEX4T1. *E. coli* BL21-Gold(DE3)pLysS cells were transformed with pGEX4T1 or pET28a expression vectors and treated with 0.1 mmol/L of isopropyl-D-thiogalactoside for 4 h. Ubiquitin was constructed by PCR and various mutated ubiquitin constructs were generated by site-directed mutagenesis. The diagram of ubiquitin mutants is as follows.

6 11 27 29 33 48 63

Ub ----K-----K----K----K------K------K-----K---

Ub(K48) ----R-----R----R----R-----R------K-----R---

Ub(K63) ----R-----R----R----R-----R------R-----K---

Ub(K63R) ----K-----K----K----K------K------K-----R---

Ub(K48R) ----K-----K----K----K------K------R-----K---

### Immuno-staining of cells

The cells, grown on glass coverslip were washed and fixed with 4% formaldehyde for 20 minutes, followed by permeabilization with 0.1% Triton X-100. After wash, the coverslips were blocked in 3% BSA for 1 h, followed by the incubation with primary antibody at 4°C overnight. After wash, the coverslips were incubated with fluorescence dye-conjugated secondary antibody for 30 min. Finally, the cells were stained with DAPI in the dark for 3 min. The stained cells were mounted and observed in A Zeiss LSM 510 META (Axiovert 200) microscope.

### Nuclear protein extraction

Cells were washed and suspended in 100 µL of buffer A (20 mM HEPES, pH 7.9, 1.5 mM MgCl_2_, 10 mM KCl, 1 mM dithiothreitol, 0.1 mM EGTA, 0.1 mM EDTA, 0.5 mM phenylmethylsulfonyl fluoride, and 0.2 mM phenylmethylsulfonyl fluoride, 0.5% Nonidet P-40). The cells were lysed, followed by centrifugation at 1700× *g* for 10 min. After separation of the cytoplasmic fraction, nuclei were washed once with Buffer A and resuspended in ice-cold buffer B (10 mM HEPES, pH 7.9, 25% glycerol, 1.5 mM MgCl_2_ and 0.4 mM NaCl, 1 mM dithiothreitol, and 0.2 mM phenylmethylsulfonyl fluoride). After 1 h incubation on ice and a centrifuge to clear the cellular debris, the nuclear extracts were collected and used for immunoblotting.

### Transient transfection

Transient transfection of cells was performed using LipofectAMINE-2000 or LipofectAMINE-PLUS (Invitrogene) as per the manufacturer's instructions.

### Immunoprecipitation and immunoblotting

Immunoblotting and immunoprecipitation was performed as described [24]. Briefly, 500 µg of cell lysates were incubated with 1 µg of primary antibody at 4°C for 3 h. Then, 20 µL of protein A/G PLUS agarose beads was added and the incubation continued overnight. The beads were washed and boiled in SDS-PAGE loading buffer for 5 minutes before electrophoresis.

### GST pull-down assay

GST pull-down assay was performed as described [24]. Bacterial cells were lysed using the following buffer: 20 mmol/L Tris-Cl, 150 mmol/L NaCl, 2 mmol/L EDTA, 0.5% NP40, pH 7.5. To determine the interaction between nCLU and pVHL, bacterial lysates containing GST-nCLU were incubated with glutathione-Sepharose 4B beads at 4°C for 1 h. The beads were washed and incubated with bacterial cell lysates containing His-pVHL, allowing the interaction between GST-nCLU and pVHL. After washing, GST-nCLU and the bound pVHL were eluted from the beads and subjected to electrophoresis.

### *In vitro* ubiquitination assay

In vitro ubiquitination assay was performed as described [25] and the Reaction II reticulocyte conjugation kit (Bostonbiochem, MA) was employed in this assay. To isolate the pVHL complex, Myc-VHL was transiently expressed in 293T cells and four hundred micrograms of the cellular proteins were immunoprecipitated with 1 µg of Myc antibody at 4°C for 3 h. Twenty microliters of protein A/G PLUS agarose beads were added and the incubation continued at 4°C overnight. The beads were washed for four times with RIPA buffer, followed by two washes with buffer containing 50 mM Tris-HCl and 3 mM DTT, pH 8.0. The agarose beads that contained purified pVHL complex were suspended in a final reaction volume of 50 µL containing ATP-regenerating buffer, 5 µg/µL ubiquitin, 1 µg/µL GST-nCLU, 100 ng/µL ubiquitin aldehyde, MG132 (5 µM), and 16 µL of purified rabbit reticulocyte fraction II, and incubated at 37°C for 2 h. After reaction, the total proteins generated in the ubiquitination reaction were analyzed by western-blot.

### Statistical analysis

The data represent the mean ± SD from three independent experiments except where indicated. Statistical analysis was performed using the unpaired two-tailed Student's *t*-test. The difference is considered significant when a *P* value is less than 0.05.

## Results

### pVHL interacts with nCLU

**Figure 1:**
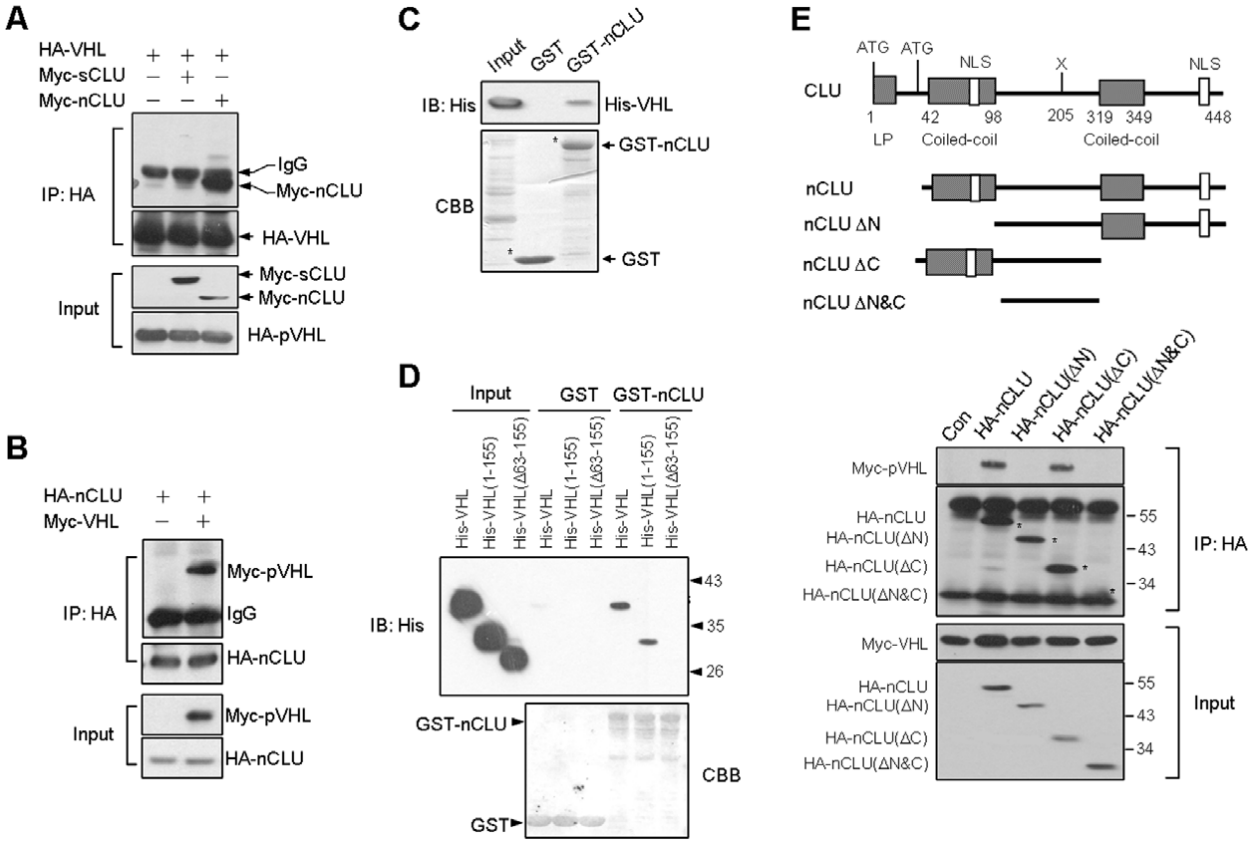
pVHL binds to nCLU. (A) 293T cells were transfected as indicated. In 24 h, the cells were harvested and cellular proteins were prepared. Immunoprecipitation was performed using HA antibody. (B) 293T cells were transfected with Myc-VHL and HA-nCLU. In 24 h, the cells were harvested and immunoprecipitation was performed using HA antibody. (C) Direct interaction between nCLU and pVHL. Equal amount of bacterial lysates containing His-pVHL were incubated with the glutathione-sepharose beads that already captured GST or GST-nCLU. The beads were washed and His-pVHL retained on beads was determined by immunoblotting. CBB, Coomassie Blue Staining. (D) nCLU bound to pVHL at β domain. Equal amount of bacterial lysates containing His-pVHL, His-pVHL(1-155) or His-pVHL(Δ63-155) were incubated with the glutathione-sepharose beads that already captured GST or GST-nCLU. The beads were washed and His-pVHL or the truncated His-pVHL retained on beads was determined by immunoblotting. (E) A various constructs encoding different nCLUs were designed. 293T cells were transfected with Myc-VHL and nCLU construct as indicated. 24 h post-transfection, the cells were harvested for immunoprecipitation using HA antibody.

pVHL is an adaptor protein and binds to many proteins. We determined whether pVHL had an interaction with sCLU and nCLU. 293T cells were transiently transfected with HA-VHL plus Myc-sCLU or HA-VHL plus Myc-nCLU plasmids. Immunoprecipitation was performed and the results indicated that HA-pVHL co-immunoprecipitated with Myc-nCLU (Fig. 1A, Fig. S1A) but not Myc-sCLU (Fig. 1A), indicating that pVHL had an interaction with nCLU. We repeated the experiment in 293T cells to confirm the interaction between pVHL and nCLU. The cells were transfected with Myc-VHL and HA-nCLU vectors. The results showed that Myc-pVHL and HA-nCLU also co-immunoprecipitated (Fig. 1B, Fig. S1B). A direct interaction between pVHL and nCLU was ascertained by GST-pull down assays (Fig. 1C). These results suggest that pVHL binds to nCLU directly.

We determined the domain of pVHL that nCLU might bind to. pVHL contains two functional sub-domains termed α and β, and the β domain (residues 63–155) functions for recognition of substrate. GST-pull down assay was performed using pVHL fragments covering different regions. VHL encodes wild-type pVHL, VHL(1-155) encodes pVHL without α domain, and VHL(Δ63-155) encodes pVHL without β domain. The results showed that pVHL and pVHL(1-155) bound to nCLU (Fig. 1D). However, pVHL(Δ63-155) had no interaction with nCLU. The results suggest that nCLU binds to the β domain of pVHL and nCLU might be a substrate of pVHL.

We next determined the site of nCLU that pVHL bound to. Our results showed pVHL had no interaction with nCLU if the N-terminal coil-coil domain of nCLU was deleted (Fig. 1E), suggesting that this domain is required for nCLU-pVHL interaction. sCLU also has the coil-coil domain, however, it does not bind to pVHL (Fig. 1A). sCLU was translated from the first ATG and nCLU was translated from the second ATG (Fig. 1E). So, the N-terminal of sCLU is different from that of nCLU. This might be the reason why nCLU binds to pVHL but sCLU does not. This needs further investigation.

### pVHL mediates ubiquitination of nCLU

**Figure 2:**
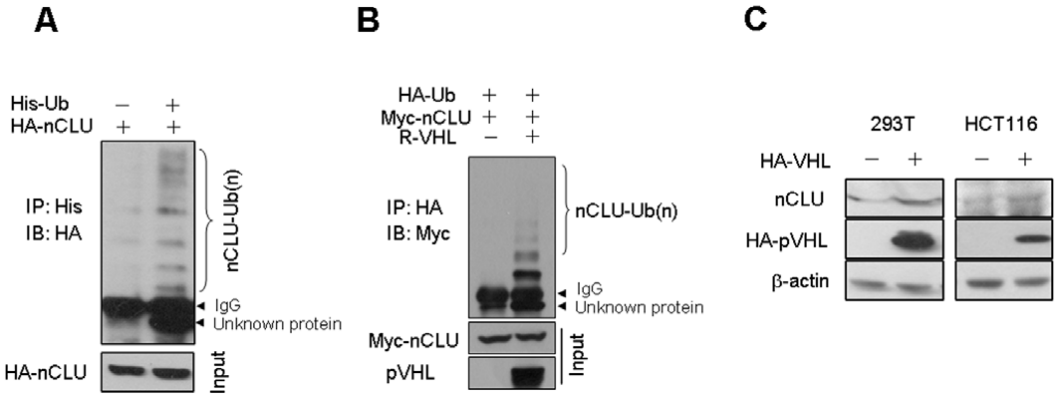
pVHL mediates ubiquitination of nCLU. (A) Modification of nCLU by ubiquitin. 293T cells were transfected with indicated plasmids and incubated for 24 h before harvest for immunoprecipitation and western-blot. (B) pVHL enhanced the ubiquitination of nCLU. 293T cells were transfected with HA-Ub, Myc-nCLU and R-VHL plasmids. In 24 h, the cells were harvested and cellular proteins were prepared for immunoprecipitation using HA antibody. (C) HCT116 and 293T cells were transfected with Myc-VHL. In 48 h, the cells were harvested and lysed. Expression of nCLU was determined by western-blot.

pVHL functions as the substrate recognition component of an E3-ubiquitin ligase that targets proteins for degradation. pVHL interacted with nCLU (Fig. 1). Previous study demonstrated that nCLU undergoes proteasomal degradation [26]. We therefore tested whether pVHL mediated ubiquitination of nCLU. We found that nCLU had polyubiquitination (Fig. 2A) and overexpression of VHL increased polyubiquitination of nCLU (Fig. 2B). The identity of the band below IgG is unknown (Fig. 2A and B). We determined whether pVHL promoted degradation of nCLU. Unexpectedly, ectopic expression of VHL didn't inhibit expression of nCLU (Fig. 2C).

### pVHL mediates K63-linked polyubiquitination of nCLU

**Figure 3:**
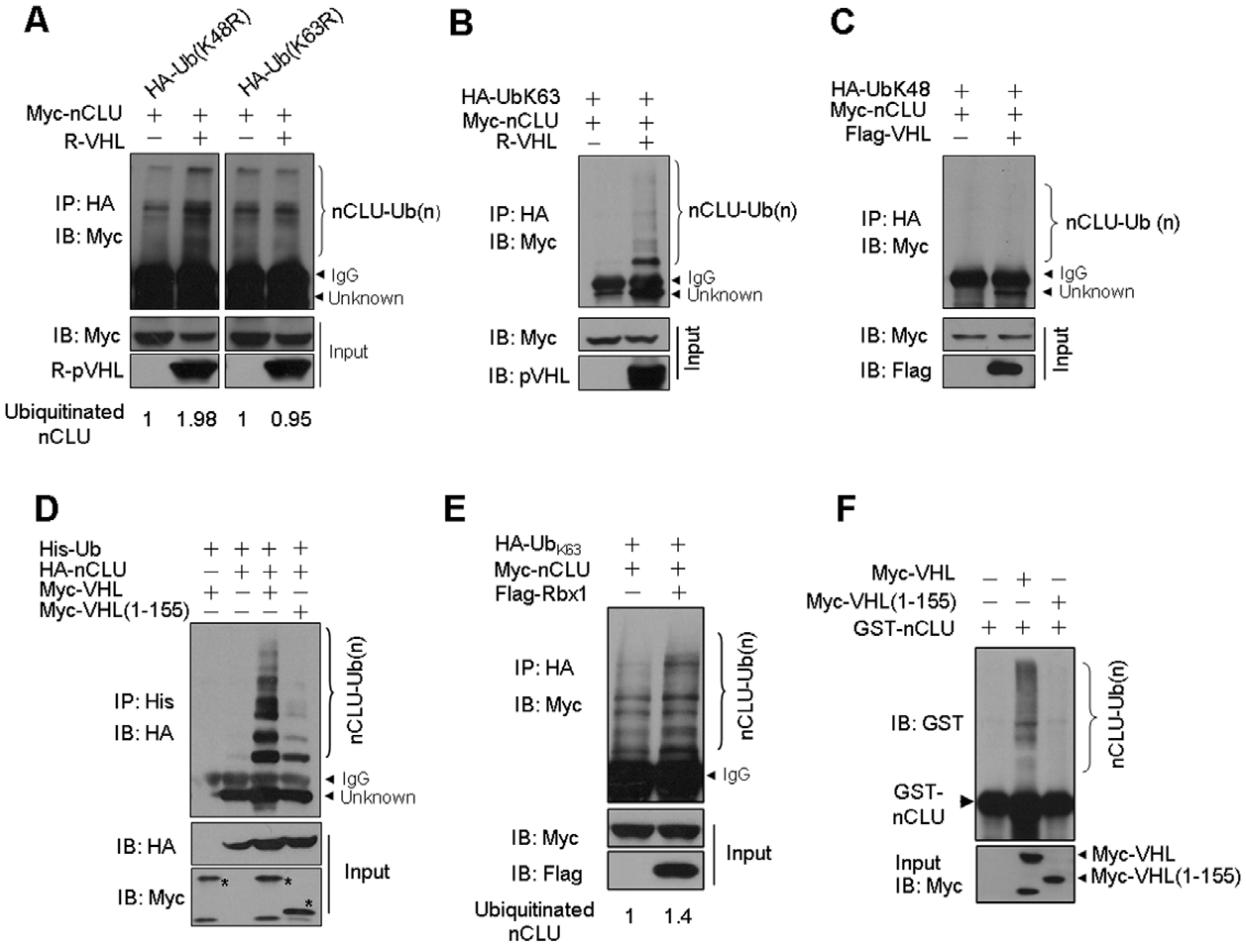
pVHL mediates K63-linked ubiquitination of nCLU. (A) pVHL enhanced the K63-linked ubiquitination of nCLU. 293T cells were transfected with Myc-nCLU, R-VHL and HA-Ub(K48R) (or Ub(K63R)). In 24 h, the cells were lysed and immunoprecipitation was done using HA antibody. The relative amount of ubiquitinated nCLU was determined by densitometry assay. (B) pVHL enhanced the ubiquitination of nCLU in the presence of Ub(K63). 293T cells were transfected with Myc-nCLU, R-VHL and HA-Ub(K63) plasmids. In 24 h, the cells were harvested and then lysed for immunoprecipitation using HA antibody. (C) pVHL did not enhance nCLU ubiquitination in the presence of Ub(K48). 293T cells were transfected with Myc-nCLU, R-VHL and HA-Ub(K48) plasmids. In 24 h, the cells were harvested and then lysed for immunoprecipitation using HA antibody. (D) pVHL(1-155) did not mediate ubiquitination of nCLU. 293T cells were transfected with various constructs as indicated. In 24 h, the cell lysates were prepared for immunoprecipitation and western-blot. (E) Overexpression of Rbx1 enhanced K63-linked ubiquitination of nCLU. 293T cells were transfected as indicated. In 24 h, the cells were harvested and cell lysates were prepared for immunoprecipitation. The relative amount of ubiquitinated Myc-nCLU was determined by densitometry assay. (F) pVHL mediated K63-linked ubiquitination of nCLU *in vitro*. *In vitro* ubiquitination assay was performed as described under Methods.

It is known that, the K48-linked polyubiquitination leads to protein proteasomal degradation and the K63-linked polyubiquitination plays a role in protein trafficking, kinase and phosphatase activation [27]. One of the examples is the non-degradative K63-polyubiquitination of NEMO that leads to NF-κB activation [28]. This drove us to determine the kind of ubiquitination of nCLU that pVHL mediated. We employed in our work two ubiquitin mutants Ub(K48R) and Ub(K63R) where K48 or K63 was mutated to arginine (R). 293T cells were transfected with nCLU, VHL and Ub(K48R) or Ub(K63R) constructs. Overexpression of VHL enhanced polyubiquitination of nCLU in the presence of Ub(K48R) (Fig. 3A). However, overexpression of VHL had little effect on the polyubiquitination of nCLU in the presence of Ub(K63R) (Fig. 3A), implying that pVHL mediates K63-linked ubiquitination of nCLU. Next, we employed a construct encoding Ub(K63) in our experiments. Ub(K63) is the ubiquitin that the lysine residues are mutated to R except K63. We found that overexpression of VHL enhanced polyubiquitination of nCLU in the presence of Ub(K63) (Fig. 3B). We also used a construct encoding Ub(K48). All lysine residues of it are mutated to R except K48. We found that overexpression of VHL did not enhance polyubiquitination of nCLU in the presence of Ub(K48) (Fig. 3C). Taken together, these results suggest that pVHL mediates K63-linked ubiquitination of nCLU.

The α domain of pVHL associates with the E3 ubiquitin ligase complex [2]. We found that the ubiquitination of nCLU was reduced significantly when VHL(1-155) was tested (Fig. 3D, Fig. S2). Because pVHL(1-155) did not have α domain, the results imply that the α domain of pVHL is required for pVHL to mediate ubiquitination of nCLU. Moreover, expression of VHL(Δ63-155) had little effect on ubiquitination of nCLU (Fig. S2).

Next, we determined the effects of Rbx1, a subunit of the pVHL E3 ubiquitin ligase complex, on K63-linked ubiquitination of nCLU. We found that ectopic expression of Rbx1 enhanced K63-linked ubiquitination of nCLU (Fig. 3E). We also determined whether pVHL could mediate K63-linked polyubiquitination of nCLU *in vitro*. The pVHL complex was isolated by immunoprecipitation and incubated with purified nCLU in Reaction II Reticulocyte Conjugation Kit as described previously [25]. We found that the isolated pVHL complex mediated K63-linked ubiquitination of nCLU *in vitro* (Fig. 3F). However, the isolated pVHL(1-155) could not mediate K63-linked ubiqitination of nCLU *in vitro*. A control experiment was done. The results showed that the ubiquitin chains were attached to GST-nCLU and not to pVHL itself and Ub(K63R) was not linked to nCLU (Fig. S3). Taken together, these results suggest that pVHL E3 ubiquitin ligase complex mediates K63-linked ubiquitination of nCLU.

### pVHL promotes nucleus localization of nCLU

**Figure 4:**
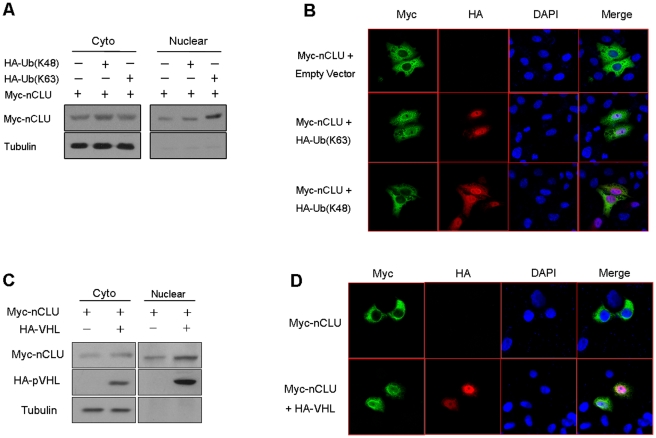
pVHL enhances nuclear translocation of nCLU. (A) Overexpression of Ub(K63) promoted nCLU nucleus translocation. 293T cells were transfected with the constructs as indicated. In 24 h, the nuclear and cytoplasmic proteins were prepared for immunoblotting. (B) Overexpression of Ub(K63) promoted nCLU nucleus translocation. Hela cells were transfected with the constructs as indicated. In 24 h, the cells were fixed and immunostained as described under methods. The pictures were obtained under a confocal microscope. (C) Overexpression of pVHL promoted nCLU nuclear translocation. 293T cells were transfected as indicated. In 24 h, the cells were harvested and nuclear and cytoplasmic proteins were prepared for immunoblotting. (D) pVHL promoted nCLU nuclear translocation. Hela cells were transfected as indicated. In 24 h, the cells were fixed and immuno-stained as described in Methods.

It is believed that nCLU functions in nucleus. We therefore asked whether the K63-linked ubiquitination promoted nuclear translocation of nCLU. To know this, we determined the effect of expression of Ub(K63) on cellular distribution of nCLU. We found that ectopic expression of Ub(K63) enhanced nuclear localization of nCLU (Fig. 4A). However, overexpression of Ub(K48) had little effect on nCLU nuclear translocation. Immuno-staining of the cells also indicated that ectopic expression of Ub(K63) enhanced nuclear localization of nCLU (Fig. 4B). It was noted that overexpression of Ub(K63)+nCLU, but not Ub(K48)+nCLU, led to alteration of cell morphology (Fig. 4B). Taken together, these results suggest that the K63-linked ubiquitination of nCLU promotes nCLU nuclear translocation.

Next, we determined the effect of pVHL on nCLU nuclear translocation. The nuclear and cytoplasmic proteins were isolated and western-blot was performed. We found that overexpression of pVHL enhanced protein levels of nCLU in nucleus (Fig. 4C). Immuno-staining of the cells was also performed to indicate nCLU distribution in cells. We found that Myc-nCLU proteins were mainly cytoplasmic when the cells were transfected with Myc-nCLU alone (Fig. 4D). If Myc-nCLU were co-transfected with HA-VHL, the Myc-nCLU proteins in nucleus were increased. These results suggest that pVHL promotes nuclear translocation of nCLU. We noted that expression of exogenous VHL+nCLU altered cell morphology (Fig. 4D). This is similar to the results of Fig. 4B. The altered cell morphology might be due to increased nCLU in nucleus.

## Discussion

It is known that pVHL targets HIFα for proteasomal degradation. As an adaptor protein, pVHL not only binds to HIFα but also binds to many other proteins and has multiple functions [1]. We found in our work that pVHL bound to nCLU and mediated nCLU K63-linked ubiquitination, which may lead to nuclear translocation of nCLU. To our knowledge, this is the first report to demonstrate that pVHL mediates K63-linked ubiquitination of a protein.

The addition of ubiquitin to proteins occurs through an enzymatic cascade. Firstly, an activating enzyme, E1, is charged with ubiquitin, then the ubiquitin is transferred to an E2-conjugating enzyme and, finally, it is attached to a substrate by an E3 ligase [29]. There are a few types of polyubiquitin chains [27] and in many conditions the type of polyubiquitin chain formed appears to be directed primarily by the cooperating E2 enzyme. E2 dictates the type of inter-ubiquitin linkages and thereby determines the fate of the substrate [30]. We found that pVHL mediated K63-linked ubiquitination of nCLU (Fig. 3) and promoted nCLU nuclear translocation (Fig. 4). Our results suggest that, for different substrates, pVHL may mediate different types of ubiquitination, probably using different E2 enzymes.

Although protein ubiquitination has been best characterized as a mechanism for targeting proteins to the proteasome for degradation, recently it has become clear that this modification can also serve many other functions. For example, K48-linked ubiquitination tags the target protein to be degraded by the proteasome, and K29-linked ubiquitination is considered as an indicator of subsequent lysosomal degradation [31]. During the last few years the K63-ubiquitination has emerged as a novel post-translational modification of remarkable functional interest for fine-tuning signal transduction pathways [27], [32]. We found that ectopic expression of pVHL promoted nuclear translocation of nCLU (Fig. 4). This might be one function of pVHL that mediates K63-linked ubiquitination of nCLU. There are currently a few examples of ubiquitin serving as a nuclear import signal. For example, TRAF6, an E3 ubiquitin ligase, was shown to mediate K63-linked ubiquitination of p75 cytoplasmic interactor (NRIF) and this modification is necessary for NRIF nuclear translocation [33].

nCLU has been found to trigger cell death when in nucleus in a few systems [22]. However, we did not find that overexpression of nCLU led to cell apoptosis in our work. The difference might be due to different cells used in these works. It should be noted that in our work the increased nucleus nCLU do altered the morphology of these cell (Fig. 4B and D). The physiological function of pVHL that mediates nCLU ubiquitination needs further investigation. For example, it will be interesting to determine whether pVHL-mediated ubiquitination and nucleus translocation of nCLU plays an important role in regulation of cell proliferation and apoptosis. Another important question is whether pVHL is required for nCLU nucleus translocation.

In this manuscript, we have disclosed a novel function of pVHL that mediates K63-linked ubiquitination and identified nCLU as a new target of pVHL. These results indicate that pVHL mediates not only K48- but also K63-linked ubiquitination of proteins. It is known that K48-linked ubiquitination usually leads to protein degradation and K63-linked ubiquitination functions as a post-translational modification for protein trafficking and fine-tuning signal transduction pathways. Our results may provide a new sight of pVHL's function.

## Supporting Information

Figure S1 (A) 293T cells were transfected with Myc-nCLU plus empty vector or Myc-nCLU plus HA-VHL. In 24 h, the cells were harvested and cellular proteins were prepared. Immunoprecipitation was performed using HA antibody. The results of negative control experiment showed that HA antibody did not interact with Myc-tagged nCLU. (B) 293T cells were transfected as indicated. In 24 h, the cells were harvested and immunoprecipitation was performed using HA antibody. The results of negative control experiment indicated that the HA antibody did not interact with Myc-tagged VHL.
(TIF)

Figure S2 **pVHL(1-155) and pVHL(Δ63-155) had little effect on ubiquitination of nCLU.** 293T cells were transfected with HA-nCLU, His-Ub and various Myc-VHL constructs as indicated. In 24 h, the cells were harvested and cellular proteins were prepared for immunoprecipitation and western-blot.
(TIF)

Figure S3 **pVHL mediated K63-linked ubiquitination of nCLU** ***in vitro*** **.** *In vitro* ubiquitination of nCLU by pVHL was performed as described under Methods. Ubiquitin or Ub(K63R) at 5 µg/µL was added to the reaction system.
(TIF)
