# Supplementary figures and images for: pVHL Mediates K63-Linked Ubiquitination of nCLU
Source: PLoS One. 2012 Apr 20;7(4):e35848. doi: 10.1371/journal.pone.0035848 (PMC3332038; doi:10.1371/journal.pone.0035848)

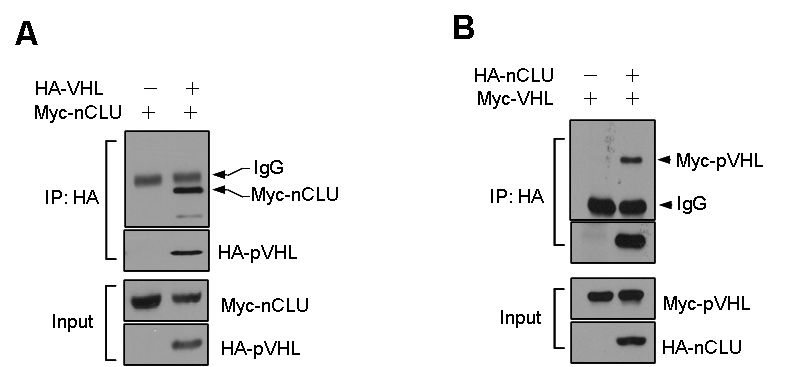

Supplement: Figure S1 — (A) 293T cells were transfected with Myc-nCLU plus empty vector or Myc-nCLU plus HA-VHL. In 24 h, the cells were harvested and cellular proteins were prepared. Immunoprecipitation was performed using HA antibody. The results of negative control experiment showed that HA antibody did not interact with Myc-tagged nCLU. (B) 293T cells were transfected as indicated. In 24 h, the cells were harvested and immunoprecipitation was performed using HA antibody. The results of negative control experiment indicated that the HA antibody did not interact with Myc-tagged VHL. (TIF) [file pone.0035848.s001.tif]

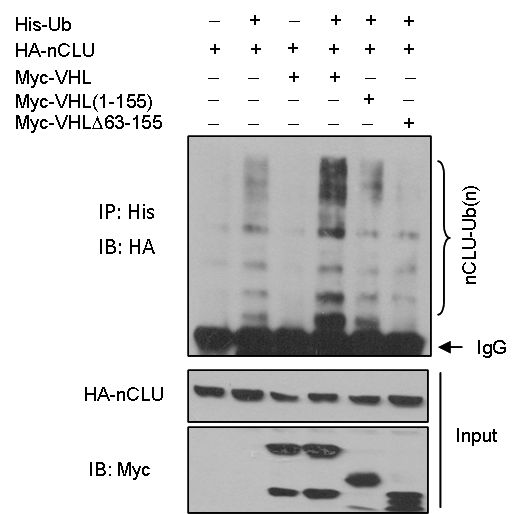

Supplement: Figure S2 — pVHL(1-155) and pVHL(Δ63-155) had little effect on ubiquitination of nCLU. 293T cells were transfected with HA-nCLU, His-Ub and various Myc-VHL constructs as indicated. In 24 h, the cells were harvested and cellular proteins were prepared for immunoprecipitation and western-blot. (TIF) [file pone.0035848.s002.tif]

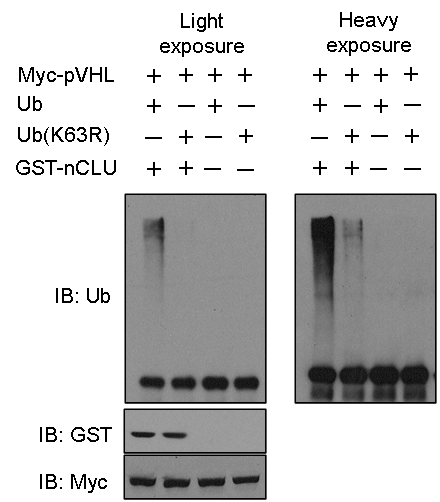

Supplement: Figure S3 — pVHL mediated K63-linked ubiquitination of nCLU in vitro . In vitro ubiquitination of nCLU by pVHL was performed as described under Methods. Ubiquitin or Ub(K63R) at 5 µg/µL was added to the reaction system. (TIF) [file pone.0035848.s003.tif]
